# Worsening heart failure is not a single endpoint: a severity-gradient perspective from digoxin in rheumatic heart disease

**DOI:** 10.1007/s10741-026-10684-6

**Published:** 2026-10-10

**Authors:** Koichiro Yuji, Wakako Yuji

**Affiliations:** 1 Project Division of International Healthcare Innovation Research, The Institute of Medical Science, the University of Tokyo, Tokyo, Japan; 2 Department of Research, Kitasato Institute Hospital, Tokyo, Japan

**Keywords:** Rheumatic heart disease, Heart failure, Digoxin, Clinical trial endpoints, Low- and middle-income countries

## Abstract

The composite of cardiovascular or all-cause death and worsening heart failure (WHF) has become the principal efficacy endpoint of heart failure trials. Yet WHF is not one event: it spans outpatient oral-diuretic escalation, urgent intravenous-diuretic visits, hospitalisation, and HF-related death—tiers that differ markedly in frequency, ascertainment, and prognostic weight. A composite WHF benefit therefore cannot be properly interpreted without knowing which tier carries the signal, and we argue that this tier is both phenotype- and setting-dependent. Two trials published on the same day in 2026 make the point with unusual clarity. In the Dig-RHD trial of digoxin in rheumatic heart disease, the benefit concentrated in outpatient diuretic events, with neutral results at hospitalisation and death; in a contemporaneous meta-analysis of digitalis glycosides in reduced or mildly reduced ejection fraction, the same drug class reduced worsening heart failure mainly through hospitalisation, again without a mortality benefit. We use this contrast to propose a severity-gradient framework for reporting and interpreting worsening heart failure, and discuss its implications for trial design, for the translation of guideline-based evidence across health systems, and for the use of inexpensive therapies such as digoxin in low- and middle-income settings.

## The endpoint that now defines heart failure trials

For three decades, all-cause mortality was the currency of heart failure (HF) therapeutics. Today the dominant efficacy signal is the composite of cardiovascular or all-cause death and worsening heart failure (WHF) [1]. The change is reasonable—foundational therapy has made mortality trials impractical, and recurrent decompensation is itself patient-important—but WHF is not one event. As operationalised across trials it spans an increase in oral diuretic dose managed in clinic, an urgent visit for intravenous diuretics, hospitalisation, and death from progressive HF: tiers that differ in frequency, ascertainment, and prognostic weight [1]. When a trial reports a benefit on a composite WHF endpoint, the decisive question is which tier produced it. That question is too often left unexamined, the answer is phenotype- and setting-dependent, and two trials published on the same day in 2026 illustrate the point.

### Worsening heart failure is a severity gradient

The events grouped under WHF form a prognostic gradient, and trials have progressively extended the endpoint down it—from hospitalisations and deaths, to urgent intravenous-diuretic visits, and most recently to outpatient oral-diuretic escalation [1]. Each broadening raises event counts and statistical power but lowers the average prognostic weight per event. Ascertainment compounds this: outpatient diuretic changes are recorded only if a trial actively solicits them, so cross-trial comparisons of a composite are partly comparisons of measurement systems. The milder tiers are nonetheless not benign: in a prespecified analysis of DAPA-HF, outpatient diuretic intensification predicted a nearly threefold rise in one-year mortality (hazard ratio [HR] 2.67; 95% CI 2.03–3.52) [2]. The meaning of each tier also varies by health system. In a global HF cohort the ratio of a first HF hospitalisation to death was 3.8 in high-income countries but 1.1 in low- and middle-income countries (LMICs) [3], because patients there reach hospital late, if at all. Where hospitalisation is inaccessible it marks access rather than severity, and the informative signal migrates to the outpatient tier.

### Digoxin in rheumatic heart disease: benefit at the bottom of the gradient

In the Dig-RHD trial, 1759 patients with symptomatic rheumatic heart disease (RHD) received digoxin or placebo [4]. Digoxin reduced the composite of all-cause death or new-onset or worsening HF (276/880 [31.4%] vs 312/879 [35.5%]; HR 0.82; 95% CI 0.70–0.97), driven by worsening HF (HR 0.82; 95% CI 0.69–0.98). The benefit sat at the bottom of the gradient: about 80% of first WHF events (385 of 484) were outpatient or intravenous diuretic escalations. At more severe tiers the signal disappeared—first HF hospitalisation did not differ (HR 1.04; 95% CI 0.70–1.54), nor did HF-related death or hospitalisation (HR 0.90; 95% CI 0.66–1.22)—and all-cause death was unchanged (HR 0.94; 95% CI 0.70–1.26). About 14 patients were treated to prevent one event over two years, with permanent discontinuation for suspected toxicity in roughly 1%.

The mechanism fits the phenotype: in multivalvular RHD with frequent atrial fibrillation and preserved ejection fraction, the rate-controlling and mildly inotropic effects of digoxin plausibly reduce congestion managed in clinic rather than reverse systolic failure. Pivotal HF trials have largely excluded significant valvular disease, so the evidence base for modern guideline-directed therapy maps poorly onto RHD; against that backdrop an old, inexpensive oral drug that reduces outpatient decompensation addresses a real and under-served need. The distribution also fits the setting—in Dig-RHD the hospitalisation-to-death ratio was about 0.6, even lower than in other LMIC cohorts [3], and 37% of patients hospitalised for HF died within 30 days, so hospitalisation acted as late rescue rather than as a sensitive marker. RHD still affects tens of millions and kills the young chiefly through heart failure [5, 6], with South Asia and sub-Saharan Africa disproportionately affected. For these patients, many without access to valve surgery or newer therapies, an inexpensive drug that reduces outpatient decompensation matters even without a mortality benefit; where hospital access is limited, preventing outpatient decompensation is a central determinant of quality of life and health-system cost.

### The same drug class, a different tier

**Fig. 1:**
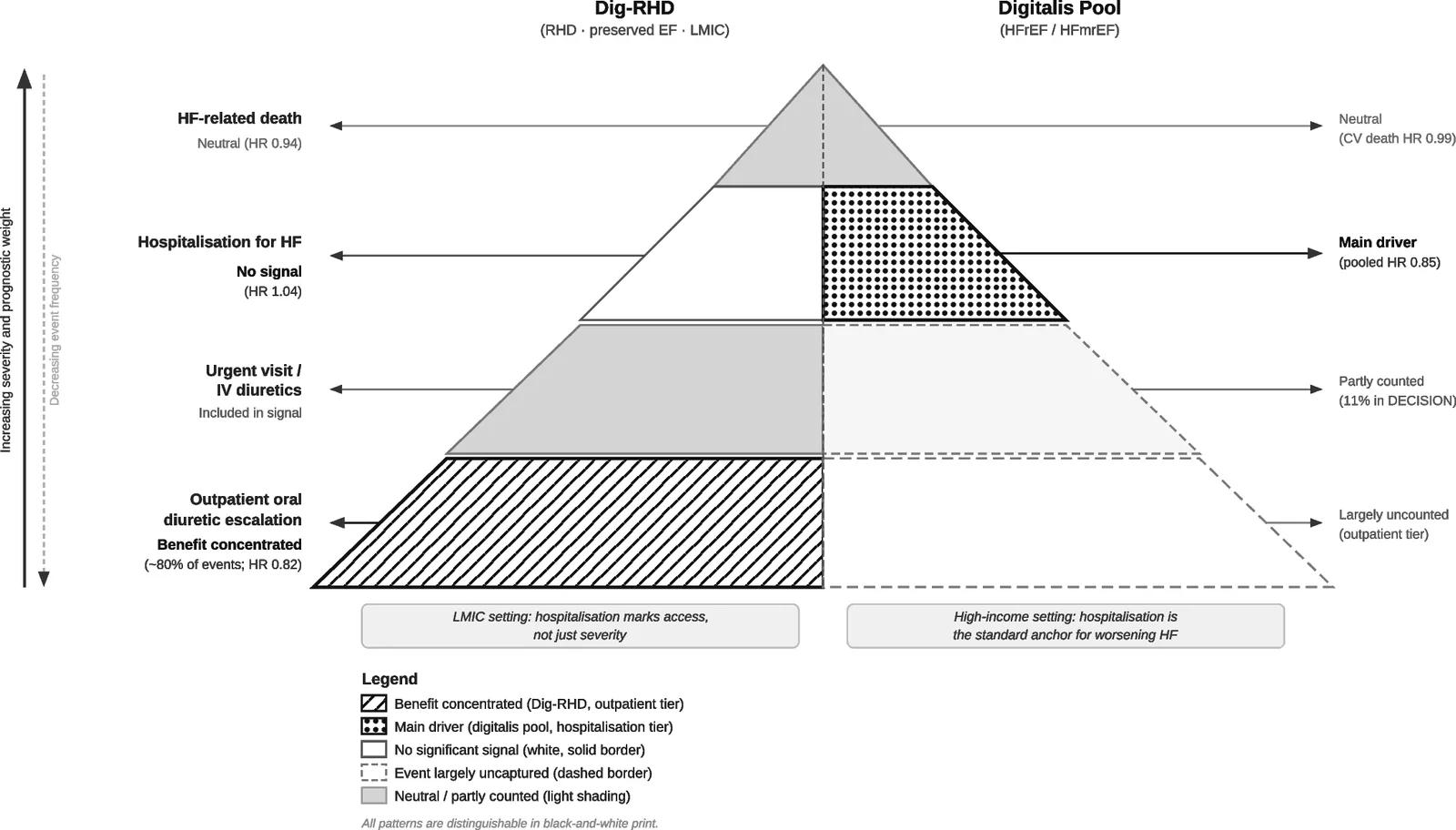
The severity gradient of worsening heart failure and the contrasting distribution of the digitalis treatment signal in two 2026 datasets. Worsening heart failure (WHF) events span a prognostic gradient depicted here as a pyramid, with the base representing the most frequent, lowest-weight tier and the apex the least frequent, highest-weight tier: from outpatient oral diuretic escalation (the broadest tier, highest frequency but lowest individual prognostic weight) to heart failure–related death (the narrowest tier, highest weight). The pyramid is split to contrast two 2026 datasets. Left (diagonal-line hatching): In the Dig-RHD trial (rheumatic heart disease, preserved ejection fraction, low- and middle-income setting), the digoxin benefit was concentrated in the outpatient tier (hatched shading), with no signal at hospitalisation (unshaded, hazard ratio 1.04). Right (dot pattern): In pooled trials of digitalis glycosides in reduced or mildly reduced ejection fraction (largely high-income settings), the benefit was driven mainly by hospitalisation (dot-pattern shading, pooled hazard ratio 0.85), while outpatient events were largely uncaptured (dashed empty outline). At the death tier, mortality remained neutral in both groups (cardiovascular death hazard ratio 0.99 for the digitalis pool). All fills and patterns are distinguishable in black-and-white print: diagonal-line hatching, dot pattern, solid white, dashed-border white, and light shading are used to differentiate signal status without relying on colour. The pyramid is a simplified conceptual model of congestion-related worsening; it does not imply a strictly stepwise clinical course, that worsening heart failure precedes all cardiac deaths, or that the two phenotypes share a common pathophysiology. CV cardiovascular, EF ejection fraction, HF heart failure, HFmrEF HF with mildly reduced EF, HFrEF HF with reduced EF, HR hazard ratio, IV intravenous, LMIC low- and middle-income country, RHD rheumatic heart disease

A companion meta-analysis published the same day pooled 9013 patients with reduced or mildly reduced ejection fraction [7]. Digitalis glycosides again reduced the composite of cardiovascular death or worsening HF (HR 0.85; 95% CI 0.80–0.90) without affecting cardiovascular (HR 0.99) or all-cause death (HR 0.97)—but, in the authors’ description, mainly through HF hospitalisation. The DIG trial had reduced HF hospitalisation with neutral mortality [8]; DIGIT-HF reduced a composite of death or HF hospitalisation [9]; and DECISION, the only trial counting outpatient urgent visits, found these were just 11% of worsening events [10]. The meta-analysis found no heterogeneity by glycoside type or era, which strengthens the present argument: if the drug effect is homogeneous, the difference in which tier carries the signal reflects the populations and the events each trial measured, not pharmacology. Across phenotypes the class signal is consistent—it reduces worsening HF, not death—but the tier at which it becomes visible differs: hospitalisation in reduced or mildly reduced ejection fraction, outpatient decompensation in RHD with preserved ejection fraction (Fig. 1). This contrast is a lens on measurement and reporting—where a class effect becomes visible under different designs and populations—not a claim that these phenotypes share a pathophysiology.

### Why the tier is not a statistical footnote

This has practical force. First, endpoint choice can determine whether an effective therapy appears useful at all: a hospitalisation-based composite—the high-income standard—would have rendered Dig-RHD null (HR 1.04), discarding a real benefit in a population with few alternatives. Second, the sensitivity to endpoint construction is visible within Dig-RHD itself: while the time-to-first-event composite was significant, hierarchical analyses that prioritise the most severe components were not (win ratio 1.17, 95% CI 0.99–1.38; net benefit 0.036, −0.006 to 0.078), because the benefit lives in the lower tiers [4]. Third, and most important for translation, applying hospitalisation-anchored high-income evidence to RHD-endemic, resource-limited practice underestimates therapies whose benefit precedes hospitalisation; the reverse mismatch leads clinicians in well-resourced systems to over-read soft composites. The same HR of 0.82 means different things to different readers: where most counted events are hospitalisations it signals fewer admissions; where most are outpatient escalations it signals fewer clinic decompensations but no change in admissions or death. A composite WHF result is interpretable only alongside the tier that produced it and the care-access profile of the population measured—and the principle extends to any therapy judged against a WHF composite, not digitalis alone.

### A proposal: report the gradient, not the composite alone

Three steps would help. Trialists should prespecify and report WHF tier by tier—event counts and the treatment effect at outpatient oral-diuretic escalation, urgent intravenous-diuretic care, hospitalisation, and HF-related death—and report the study population’s hospitalisation-to-death ratio as context; individual-patient-data meta-analyses of the digitalis trials, stratified by tier and phenotype, could then test whether the class effect is truly uniform once measurement is harmonised. Guideline writers and regulators should read a composite WHF benefit in light of its driving tier and the population’s access to care before extrapolating across settings, and reporting standards could ask trials to disclose the tier distribution of the primary endpoint. For clinicians in RHD-endemic and other LMIC settings, the Dig-RHD data support the judicious use of digoxin in symptomatic RHD to reduce outpatient worsening, with no mortality penalty and little toxicity. Table 1 gives a tier-resolved template and maps where the signal concentrated in the two datasets, and Fig. 1 depicts the same contrast.

**Table 1:** The severity gradient of worsening heart failure (WHF) and the contrasting distribution of the digitalis treatment signal in two 2026 datasets

WHF Tier (Increasing Severity)	Ascertainment & Prognostic Weight	Dig-RHD Trial^a^ *(LMIC, RHD, preserved EF)*	Digitalis Pool & DECISION Trial^b^ *(High-income, HFrEF/HFmrEF)*
Outpatient oral diuretic escalation	*Ascertainment:* Clinic visits *Weight:* Lower severity but non-benign (~ threefold increased 1-year mortality risk^c^)	**Dominant signal** Constituted ~ 80% of first WHF events; drove the overall composite benefit	**Largely uncaptured** Rarely systematically counted in legacy or contemporary pooling
Urgent visit/intravenous diuretics	*Ascertainment:* Emergency department or day-care *Weight:* Intermediate severity	**Included in signal** Captured collectively as part of the outpatient-tier composite benefit	**Minor contribution** Accounted for only 11% of events in the DECISION trial
Hospitalisation for HF	*Ascertainment:* Inpatient admission *Weight:* High severity (strongly influenced by health care access in LMICs^d^)	**No signal** No significant between-group difference (HR 1.04)	**Dominant signal** Main driver of the overall composite benefit (pooled HR 0.85)
Death (Cardiovascular/All-cause)	*Ascertainment:* Records and endpoint adjudication *Weight:* Highest severity	**Neutral** No significant effect (within HF death or hospitalisation, HR 0.90)	**Neutral** Mortality unchanged (cardiovascular death pooled HR 0.99)

*EF* Ejection fraction, *HF* Heart failure, *HFmrEF* HF with mildly reduced EF, *HFrEF* HF with reduced EF, *HR* Hazard ratio, *LMIC* Low- and middle-income country, *RHD* Rheumatic heart disease, *WHF* Worsening heart failure.

^a^Data from Karthikeyan et al. [4].

^b^Data from Damman et al. [7] and van Veldhuisen et al. [10].

^c^Prognostic risk established in the DAPA-HF trial [2].

^d^Contextualised by the G-CHF registry demonstrating marked global variations in hospitalisation-to-death ratios [3].

### Limitations

Several caveats bound this argument. First, the gradient is a deliberate simplification centred on congestion. Worsening heart failure in reduced or mildly reduced ejection fraction is often managed not by diuretics alone but by up-titrating disease-modifying therapy—beta-blockers, renin–angiotensin–aldosterone system inhibitors, angiotensin receptor–neprilysin inhibitors, sodium–glucose cotransporter-2 inhibitors, and device therapy such as cardiac resynchronisation—as well as by managing comorbidities such as anaemia and nutrition and by other non-pharmacological measures; progression from outpatient escalation to hospitalisation is frequently non-sequential. Second, worsening heart failure does not capture all cardiac death: a large share of deaths in reduced ejection fraction are sudden arrhythmic events not preceded by decompensation, making the endpoint an imperfect surrogate for mortality in that phenotype. Third, the pathophysiologies differ—valvular pressure and volume overload driving congestive progression in rheumatic heart disease versus the heterogeneous substrate of non-rheumatic reduced ejection fraction—so the two datasets are not biologically equivalent. Our contrast is therefore a framework for how the same drug class is measured and reported across settings, not a claim that these phenotypes lie on one biological axis.

## Conclusion

Worsening heart failure, as operationalised in trials, is better read as a gradient than as a single point. Within the limits noted above—and recognising that its tiers reflect congestion rather than the whole of heart failure progression, and that their meaning is phenotype- and setting-dependent—reporting and interpreting it tier by tier would help guard against two opposite errors: under-recognising effective, inexpensive therapies in low-resource settings, and over-reading soft endpoints in well-resourced ones. The Dig-RHD investigators, and those of the trials behind the accompanying meta-analysis, are well placed to begin—by reporting the effect of digitalis across the full severity gradient of worsening heart failure.

## Data Availability

No datasets were generated or analysed during the current study.
